# Role of Polyamines and Hypusine in β Cells and Diabetes Pathogenesis

**DOI:** 10.3390/metabo12040344

**Published:** 2022-04-12

**Authors:** Abhishek Kulkarni, Cara M. Anderson, Raghavendra G. Mirmira, Sarah A. Tersey

**Affiliations:** Department of Medicine, The University of Chicago, Chicago, IL 60637, USA; abhikulkarni@uchicago.edu (A.K.); caraa@uchicago.edu (C.M.A.); mirmira@uchicago.edu (R.G.M.)

**Keywords:** polyamines, putrescine, spermidine, spermine, hypusine, β cells, diabetes, eIF5A

## Abstract

The polyamines—putrescine, spermidine, and spermine—are polycationic, low molecular weight amines with cellular functions primarily related to mRNA translation and cell proliferation. Polyamines partly exert their effects via the hypusine pathway, wherein the polyamine spermidine provides the aminobutyl moiety to allow posttranslational modification of the translation factor eIF5A with the rare amino acid hypusine (**hy**droxy **p****u**trescine ly**sine**). The “hypusinated” eIF5A (eIF5A^hyp^) is considered to be the active form of the translation factor necessary for the translation of mRNAs associated with stress and inflammation. Recently, it has been demonstrated that activity of the polyamines-hypusine circuit in insulin-producing islet β cells contributes to diabetes pathogenesis under conditions of inflammation. Elevated levels of polyamines are reported in both exocrine and endocrine cells of the pancreas, which may contribute to endoplasmic reticulum stress, oxidative stress, inflammatory response, and autophagy. In this review, we have summarized the existing research on polyamine-hypusine metabolism in the context of β-cell function and diabetes pathogenesis.

## 1. Introduction

Diabetes is characterized by dysfunction and/or death of insulin-producing islet β cells, leading to hyperglycemia [1]. Despite intense research efforts to identify the underlying molecular mechanisms of this complex disease, there are still gaps in our understanding of the factors and pathways causing the disorder [2]. The pathways that regulate normal β-cell function are requisite to understanding the derangements inherent to dysfunctional β cells [3]. Several molecular processes have been implicated in β-cell dysfunction and/or death. These include endoplasmic reticulum (ER) stress, oxidative stress, autophagy, and inflammation [4,5,6,7]. These processes are part of normal cellular responses to stress and are activated as adaptive mechanisms to restore cellular homeostasis. However, under conditions of unmitigated cellular stress, the prolonged activation of these processes may lead to de-differentiation, dysfunction, and eventual death of β cells [8,9]. Therefore, factors and pathways that chronically promote these processes have been targets of study for the treatment of diabetes.

Polyamines are biogenic amines that are known to be involved in cell survival, cell proliferation, and protein synthesis [10]. Importantly, polyamines are also crucial precursors in the formation of the hypusine posttranslational modification of the translation factor eukaryotic translation initiation factor 5A (eIF5A), which is required for all its known functions as a translation factor [11,12]. Although the majority of the literature on polyamines has been in the context of tumorigenesis, the literature on the role of polyamines in β cells has been increasing in recent years. In this article, we review the literature that implicates a role for polyamines and the downstream hypusine modification in β-cell dysfunction and diabetes pathogenesis (see Table 1 for an overview).

## 2. Polyamines in Diabetes Pathogenesis

### 2.1. Biosynthesis and Regulation of Polyamines

Polyamines are cationic compounds containing more than one amine group. The primary biogenic polyamines relevant to this review are putrescine, spermidine, and spermine. Their positive charge at physiologic pH enables polyamines to interact strongly with negatively charged molecules, such as DNA and RNA [28,29,30,31,32]. It has been demonstrated that polyamines directly influence DNA replication [33,34], gene transcription [35,36,37,38,39], and mRNA translation [40,41,42]. Notably, rapidly growing cells exhibit higher levels of polyamines compared to quiescent cells [43,44,45]. During transcription, polyamines participate in every step of synthesis and metabolic fate of RNA [30,46]. In protein translation, polyamines influence protein biosynthesis by regulating ribosome assembly [47,48], translational fidelity [49], and translational initiation and elongation [50]. Translational regulation by the polyamines is also influenced via the hypusine modification of eIF5A (detailed in Section 3) [51].

**Figure 1:**
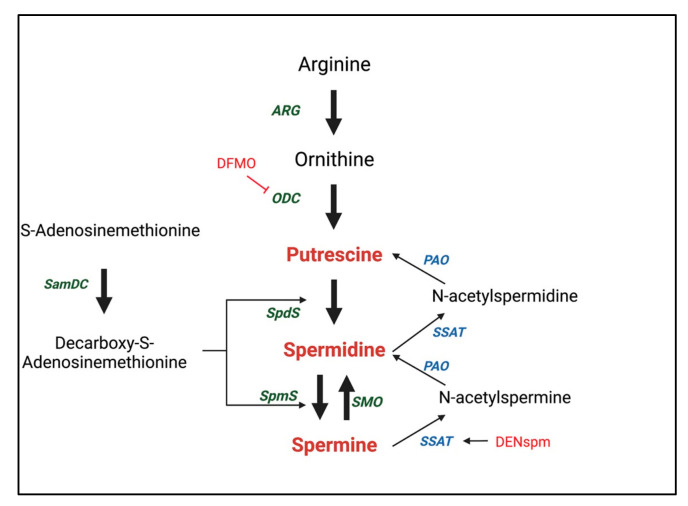
**Polyamines Biosynthesis and Catabolic Pathway**. Polyamine biosynthesis begins with arginine, where arginase (ARG) converts arginine to ornithine. Next, ornithine decarboxylase (ODC) converts ornithine to putrescine. S-adenosylmethionine decarboxylase (SamDC) then catalyzes the decarboxylation of S-adenosylmethionine. Next, spermidine synthase (SpdS) then transfers the aminopropyl moiety from the decarboxylated S-adenosylmethionine to putrescine, converting it into spermidine. Finally, spermine synthase (SpmS) catalyzes a similar aminopropyl transfer activity from the decarboxylated S-adenosylmethionine to convert spermidine to spermine. Polyamine catabolism is catalyzed by spermine/spermidine N1-acetyltransferase (SSAT) and N1-acetylpolyamine oxidase (PAO). SSAT can acetylate spermine and spermidine to N-acetylspermine and N-acetylspermidine, respectively. These acetylated products can be cleaved by PAO into spermidine and putrescine along with a generation of H_2_O_2_. Spermine can also be oxidized to spermidine by the enzyme spermine oxidase (SMO). For studying the polyamines pathway, two commonly used drugs include difluoromethylornithine (DFMO) which irreversibly inhibits ODC, and N1,N11-diethylnorspermine (DENspm) which activates SSAT.

Polyamines originate from both exogenous and endogenous sources. Exogenous sources include dietary uptake [52] and gut microbiota [53]. Endogenous sources include intracellular de novo synthesis and the interconversion of other biomolecules [54]. Endogenous polyamines biosynthesis (Figure 1) begins with the amino acid arginine. Through the action of the enzyme arginase, arginine is converted to ornithine, the precursor of major polyamines [55]. Next, ornithine decarboxylase (ODC) catalyzes the conversion of ornithine to putrescine [56]. ODC is one of the rate-limiting enzymes in polyamine biosynthesis and has a very short half-life (~10 min) [57,58]. The second rate-limiting enzyme is S-adenosylmethionine decarboxylase (SamDC), which catalyzes the decarboxylation of S-adenosylmethionine [59]. This decarboxylated S-adenosylmethionine is then used as an aminopropyl group donor. The enzyme spermidine synthase catalyzes the transfer of the aminopropyl group from decarboxylated S-adenosylmethionine to putrescine, converting it into spermidine. Spermine synthase then further catalyzes the conversion of spermidine to spermine by its aminopropyl activity, similar to that of spermidine synthase [10]. Polyamine catabolism is catalyzed by spermine/spermidine N1-acetyltransferase (SSAT) and N1-acetylpolyamine oxidase (PAO) [60]. Furthermore, spermine can be oxidized to spermidine by the enzyme spermine oxidase (SMO) [61]. In addition to the conversion pathway, a catabolic branch of polyamine metabolism exists, in which the enzyme diamine oxidase (DAO) catalyzes the polyamine oxidative deamination [62].

Polyamine metabolism is tightly regulated by the activities of ODC, SamDC, and SSAT, enzymes that are controlled through differing mechanisms. ODC activity is increased in response to growth factors, changes in the abundance of amino acids, and hypotonic stress [63,64,65]. ODC production is regulated at the transcriptional as well as the translational level. The post-translational control of ODC occurs by the induction of ornithine decarboxylase antizyme (OAZ). Accumulation of polyamines triggers OAZ, which complexes with ODC to alter its conformation, resulting in its enzymatic inhibition and acceleration of its catabolism [66]. High concentrations of polyamines also negatively regulate SamDC levels at both transcriptional and translational levels [67]. Finally, SSAT is induced by the increase in the intracellular polyamine content [68], promotes the release of polyamines in the acetylated form, and enhances putrescine oxidation by DAO [69]. Polyamines can be also be transported across the membrane by polyamine transporters [70,71]. Whereas polyamine transporters are well characterized in lower organisms, more studies in mammalian cells are warranted.

### 2.2. Polyamines in β-Cell Function and Diabetes Pathogenesis

The role of polyamines in β cells and diabetes pathogenesis is multifaceted, affecting a range of relevant biological pathways. In the pancreas, elevated levels of polyamines are observed in both exocrine and endocrine cells, with insulin-producing β cells exhibiting the highest concentrations [72,73]. Furthermore, it has also been demonstrated that polyamines have a vital role in the growth and differentiation of the pancreas [74]. Specifically in the islets, polyamines are found in the secretory granules of the β cells, where they have been associated with proinsulin biosynthesis and secretion of insulin [75]. In terms of disease pathogenesis, in murine models, it has been shown that islet polyamine levels are diminished with age and obesity [76], suggesting that alterations in intracellular polyamine levels could alter β-cell function.

#### 2.2.1. Polyamines in β-Cell Replication

Polyamines can directly affect cellular replication due to their involvement in DNA replication [33,34], gene transcription [35,36,37,38,39], and mRNA translation [40,41,42]. In the past decade, zebrafish have emerged as an attractive in vivo model for studying pancreatic development, β-cell replication, β-cell stress, and diabetes pathogenesis [77,78,79,80]. The role of polyamines during β-cell regeneration was studied using a larval zebrafish model that was treated with the irreversible inhibitor of ODC, difluoromethylornithine (DFMO) [13]. This study suggests that although polyamines are essential during pancreatic organogenesis, as also shown by a previous paper [74], polyamine biosynthesis either reduced β-cell replication or diminished the ability of the neighboring endocrine cells to trans-differentiate into β cells after β cell loss. However, it is critical to note that this study was performed in larval-stage zebrafish with a higher capacity for regeneration than adults. Hence, studies with a more mechanistic insight into the role of polyamines in cellular regeneration in adult zebrafish and/or more complex organisms are necessary to dissect the role of polyamines in β-cell regeneration. In the context of mammalian models, it has been demonstrated that the polyamines control β-cell replication via regulating c-Myc activity [14]. Specific roles of individual polyamines in β-cell replication can be potentially exploited for accurate targeting of the cell proliferation pathway and for regulating the level of proliferation induction and restoring the β-cell mass.

#### 2.2.2. Polyamines in Glucose Homeostasis

A relationship has been observed between elevated glucose levels, as seen in frank diabetes, and polyamine levels. In the rat islets, chronic treatment (24–48 h) in vitro with high glucose concentrations (20 mM) significantly elevates the production of the major polyamines putrescine and spermidine [73]. Similarly, there is an elevated synthesis of spermine in mouse islets treated with 16.7 mM glucose concentration for 48 h [81,82]. Although these differences in specific polyamine content can be attributed to the variations in species, these studies underscore the alterations in polyamine levels in the presence of high glucose conditions. Conversely, it has been demonstrated that dysregulation of polyamine metabolism can alter glucose homeostasis [15,83,84,85]. The depletion of putrescine, spermidine, and spermine in isolated mouse islets is associated with impaired glucose-stimulated insulin secretion, insulin content, insulin transcription, and DNA replication [81,82]. In vivo, it was shown that transgenic mice overexpressing SSAT, the enzyme regulating polyamine catabolism, exhibit depletion of spermidine and spermine [15]. This alteration in polyamine levels leads to impaired glucose-stimulated insulin secretion. Similarly, in mice, it has been shown that treatment with DENspm, a pharmacological agent which activates SSAT, promotes insulin resistance upon aging [85]. Whereas these studies provide evidence of the bidirectional regulation between polyamine levels and glucose homeostasis, the mechanisms underlying this relationship remain to be elucidated. One perspective that has emerged in the literature is that polyamines maintain the epigenetic landscape of specific genes via alteration of tricarboxylic acid (TCA) cycle intermediates [86]. Whether such genes and/or the translation of their encoded proteins directly affect insulin secretion or insulin sensitivity remains to be elucidated.

#### 2.2.3. Mechanistic Insight in the Role of Polyamines in Diabetes

Inflammation is a shared characteristic in both of the major forms of diabetes [87,88]. Pathogenic roles of macrophages [79,89,90,91,92,93] and T-cells [94,95,96,97,98,99] are well characterized in T1D and T2D. Polyamines are known to regulate the polarization of macrophages, which is a critical component in the regulation of inflammation [100]. Similarly, it has also been demonstrated that polyamine metabolism regulates T-cell differentiation [86,101]. These studies underscore the importance of polyamines in controlling cellular inflammatory responses. In pancreatic islet inflammation, it has been shown that the treatment of rat islets or rat insulinoma (RIN) cells with the proinflammatory cytokine IL-1β reduces the cellular content of spermidine and spermine and causes a reduction in cellular replication [102]. Another study demonstrated that IL-1β treatment promotes ODC activity in RIN cells, as confirmed by the cellular upregulation of putrescine content [103]. These studies highlight how inflammatory stimuli can alter individual polyamine levels. Targeted studies focused on the different inflammatory pathways and their effects on polyamine biosynthesis, and vice versa, would provide a complete picture of the role of polyamines in the regulation of inflammation in the context of diabetes pathogenesis.

Another potential mechanism of polyamine function in β cells is calcium regulation. In mouse β-TC6 cells, the inhibition of spermidine synthesis lead to a reduced glucose-stimulated insulin secretion (GSIS), which is associated with the inhibition of the rise of cytoplasmic Ca^2+^ concentration [104]. On the other hand, treatment of islets from a model of T2D, the db/db mouse, with spermine decreased the free-Ca^2+^ concentration through stimulation of mitochondrial Ca^2+^ uptake and mitigated the effect of inositol 1,4,5-trisphosphate (IP3) [105]. As IP3 is known to release Ca^2+^ from the ER, these results suggest that the ER and mitochondria interact with the Ca^2+^ concentration in the cytoplasm of the β cell. It is also well documented that pancreatic β cells are subject to ER stress due to Ca^2+^ deregulation in conditions of constant insulin demand [106]. Hence, it is yet to be determined whether polyamines can affect the ER stress pathway via calcium regulation or other mechanisms.

#### 2.2.4. Polyamines in Obesity and T2D

In the obesity-induced diabetes model, with respect to polyamine levels, it has been demonstrated that spermine is downregulated and spermidine unchanged in pancreatic islets [76]. Similarly, in the context of human obesity, it has been observed that polyamine levels in the blood are significantly higher in obese children than in non-obese controls [107]. It has also been reported that there is a positive correlation between serum polyamine levels and type 2 diabetes (T2D) incidence in a cohort of subjects with metabolic syndrome [20]. Specifically, it was determined that serum putrescine levels were significantly elevated in individuals with T2D and that they correlated with glycosylated hemoglobin. Moreover, serum spermine was positively correlated with fasting insulin levels [20]. However, whether the rise in serum polyamine levels is a cause or consequence of the disease remains to be determined.

#### 2.2.5. Polyamines in T1D

Studies have demonstrated that polyamines may play a pathogenic role in type 1 diabetes (T1D) disease progression. T1D is an autoimmune disease, and polyamines are known to play a critical role in the functioning of both the following major players in T1D pathogenesis: the β-cells [108] and immune cells [109]. In the non-obese diabetic (NOD) mouse model of spontaneous T1D, it was shown that inhibition of polyamine biosynthesis using DFMO significantly delays diabetes incidence, with reduced insulitis [17]. Conversely, in a recent study, it was demonstrated that spermidine supplementation resulted in enhanced diabetes incidence in the NOD mice with an increased proportion of pro-inflammatory T-cells [25]. It has also been observed that in children with T1D, there is a higher PAO activity, which could induce an increased production of free radicals and subsequent oxidative damage [18]. Similarly, the activity of spermidine oxidase, another polyamine catabolic enzyme, is significantly lower in individuals with T1D compared to non-diabetic individuals [19]. This emphasizes the fact that reduced activity of polyamine catabolic enzymes in T1D individuals might promote polyamine accumulation in the cells, leading to pathogenic conditions. In the context of human studies, there has been a clinical interest (NCT02384889) in elucidating the effect of polyamine inhibition in subjects with recent onset T1D. In this study, the subjects will be administered with different dosages of DFMO and followed up for monitoring β-cell function as well as biomarkers of β-cell stress. These studies have the potential to pave the path for novel therapeutics. Overall, in T1D conditions, the accumulation of polyamines seems to be pathogenic in terms of β-cell function as well as enrichment of pro-inflammatory immune cells.

#### 2.2.6. Effects of Polyamine Supplementation

Although polyamine accumulation is considered pathogenic to β-cell function, reduced levels of certain polyamines have been associated with diabetes pathogenesis. Studies on islet cells revealed reduced expression of spermine in the islet cells of T2D donors as compared to non-diabetic donors [20]. It is unclear if these reduced levels are causative or responsive in nature. In mouse models of T2D, a high-dose daily administration of polyamines has been shown to be an effective strategy for improvement in the metabolic health of the animal. For example, spermidine supplementation results in a significant decrease in body weight, improved glucose tolerance, and enhanced insulin sensitivity in high-fat diet-induced obese mice [22,110]. Furthermore, treatment with exogenous spermine has been shown to decrease body weight and fasting glucose and improve glucose tolerance in diet-induced obese mice [23]. Similarly, exogenous administration of spermidine in rats with pharmacologically induced diabetes improved glycemia and caused a concomitant reduction of glycosylated HbA1c levels [111]. In another similar study using the same rat model, the administration of arginine, putrescine, spermidine, or spermine was also associated with β-cell protection [112]. In a streptozotocin-mediated diabetes rat model, spermine administration did not affect hyperglycemia but improved the lipid profile and reduced the formation of advanced glycation end-products [113]. However, as mentioned earlier, spermidine treatment in the NOD mice shows enhanced diabetes incidence [25]. Whether spermidine supplementation improves the metabolic health of T1D subjects remains to be seen. Finally, spermidine administration has also been shown to modulate nitric oxide levels via the activation of autophagy [114]. These data further emphasize the effect of endogenous versus exogenous sources of polyamines. A multiomic analysis of islets treated exogenously with different polyamines could clarify the pathways altered by the polyamines.

## 3. Hypusine and eIF5A Pathway in β-Cell Function and Diabetes Pathogenesis

### 3.1. Hypusine and eIF5A Mechanistic Pathways

Protein translation is one of the key processes that are essential for maintaining cellular function. In this regard, β-cell energetics are largely expended toward protein synthesis to meet insulin production demands. In addition to the endoplasmic reticulum stress-induced by excessive translation of insulin to achieve glycemic control, β-cell health is also affected by the translation of inflammatory signaling components and proteins that influence apoptosis and the cell cycle.

**Figure 2:**
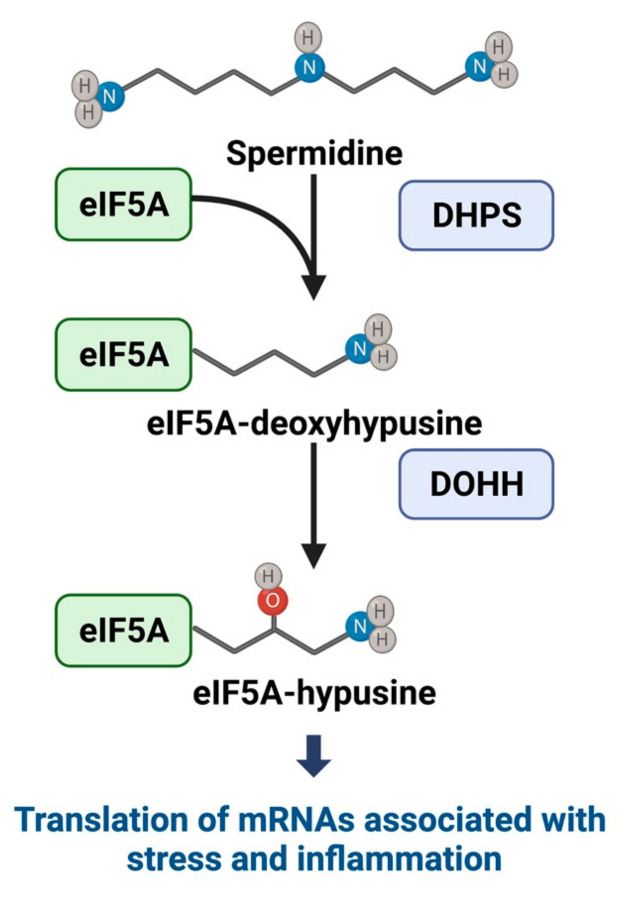
**Hypusine Pathway**. Spermidine is used as a substrate to form deoxyhypusine by the rate-limiting enzyme deoxyhypusine synthase (DHPS), which then transfers deoxyhypusine to eIF5A. Deoxyhypusine is then converted to hypusine by deoxyhypusine hydroxylase (DOHH), resulting in hypusinated eIF5A.

The translation factor eIF5A facilitates, in part, the translation of polyproline-containing peptides [115]. The functional activation of eIF5A is intimately connected with polyamine metabolism. The polyamine spermidine is used as a substrate to form deoxyhypusine by the rate-limiting enzyme deoxyhypusine synthase (DHPS). Deoxyhypusine is then hydroxylated by deoxyhypusine hydroxylase (DOHH) to form the amino acid hypusine (hydroxyputrescine lysine) (Figure 2). The only known protein containing the hypusine modification is eIF5A, which is highly conserved in all eukaryotes. DHPS catalyzes the transfer of deoxyhypusine to a conserved lysine residue on eIF5A, and a subsequent modification by DOHH forms eIF5A^hyp^ [51,116]. A second isoform of eIF5A, eIF5A2, is also encoded in the vertebrate genome and is similarly hypusinated. Though eIF5A2 shares 84% sequence homology with eIF5A, it does not appear to be constitutively expressed in all cell types [117], and its specific role(s) remain to be fully elucidated.

Hypusination of eIF5A is required for its known function as a translation factor; impaired hypusination by DHPS inhibition results in a translation initiation block [50]. eIF5A was initially identified as an initiation factor [118], but was later shown to be necessary for translation elongation [119], and termination [11]. Hypusinated eIF5A (eIF5A^hyp^) binds to stalled ribosomes at the empty E site to facilitate translation elongation [119]. Despite the necessity of eIF5A^hyp^ in translating the polyproline-containing peptides, evidence shows that it is broadly required for translation elongation and termination [12]. It is also stimulates peptidyl-tRNA hydrolysis in conjunction with eRF1, which is necessary for translation termination by peptide release [120]. Owing to their critical roles in regulating protein synthesis, eIF5A and hypusine are ubiquitous in every eukaryotic cell type and are required for many cellular processes [11,12,121]. Here, we discuss the established roles of eIF5A^hyp^ in β-cell function and diabetes pathogenesis.

### 3.2. eIF5A^hyp^ in Diabetes and the β Cell

Cellular eIF5A^hyp^ levels have apparent implications for the pathogenesis of diabetes. Recently, it was demonstrated that eIF5A^hyp^ is expressed in pancreatic tissues obtained from individuals with T1D and T2D [122]. Studies in rodent models show that eIF5A^hyp^ is essential for proper development of the exocrine pancreas [123] as well as endocrine function [14], indicating that a scarcity of eIF5A^hyp^ has detrimental consequences. However, an overabundance of eIF5A^hyp^ exacerbates the hallmarks of the diabetic phenotype, including impaired insulin secretion, reduced glucose tolerance, and insulitis. In NOD mice, pharmacological inhibition of DHPS by administration of N1-guanyl-1,7-diaminoheptane (GC7) during the prediabetic stage resulted in improved glucose tolerance, greater insulin secretion, decreased immune infiltration of islets, and a delay of diabetes onset [26,27]. Similarly, siRNA knockdown of eIF5A prevented hyperglycemia and maintained insulin secretory capacity in mice treated with low doses of streptozotocin to induce β-cell damage [16]. In the db/db mouse model of T2D, treatment with GC7 resulted in improved glucose tolerance and insulin secretion [21]. However, contrasting effects of DHPS loss have been documented in models of T2D, such as high-fat diet (HFD)-fed mice. After 4 weeks of HFD, mice with a tamoxifen-inducible β cell-specific knockout of DHPS exhibited impaired glucose tolerance and reduced insulin secretion [14]. The systemic DHPS inhibition by GC7 administration may result in a phenotype distinct from that of a β cell-specific DHPS knockout. Furthermore, structurally, GC7 itself is a polyamine that can have independent effects beyond inhibition of DHPS. Hence, the concentration of GC7 is an important factor while designing studies to ensure target-specific effects. Studying changes in the levels of eIF5A^hyp^ in different cells and tissues involved in diabetes pathogenesis may be able to explain this disparity and lead to a more nuanced understanding of the physiological mechanisms of diabetes pathogenesis. Some of these important players, including alterations in β-cell mass and immune cell response, have been investigated to explain the phenotypic consequences of upsetting the balance of eIF5A^hyp^ levels.

An expansive body of literature supports the notion that eIF5A^hyp^ is integral to cellular proliferation, specifically that cell division is inhibited without a sufficient abundance of eIF5A^hyp^ [124,125]. In mice, an adaptive proliferative response of β cells is observed during states of metabolic stress, particularly obesity, high fat feeding, and insulin resistance. This adaptation persists until just prior to the onset of T2D, at which point the β cell fails and diabetes develops [126,127,128]. Additionally, in T1D human islets, residual β-cell replication post-autoimmune attack has been observed [129], and in NOD mice, the proliferation of β cells is increased prior to the onset of diabetes [130,131]. This transient increase in β cell replication during metabolic stress is a topic of interest that may contribute to the understanding of diabetes pathogenesis.

A recent study has found that eIF5A^hyp^ in β cells is instrumental in this facultative replication response. Mice fed a high fat diet (HFD) for one week exhibited greater β-cell mass than their normal chow-fed counterparts. When DHPS is knocked out in β cells, however, this expansion was not observed after one week of HFD. eIF5A^hyp^ levels are elevated in wild-type mice fed with HFD, indicating that eIF5A^hyp^ is instrumental in compensatory β-cell replication during high fat feeding [14]. This finding has implications for potential therapeutic approaches to restoring and protecting β cell mass, and further study is required to confirm this association and understand how eIF5A hypusination can be modulated to control β cell abundance in prediabetic animals.

### 3.3. Involvement of eIF5A in Islet Inflammation and Diabetic Immune Response

Several aspects of the immune response in diabetes pathogenesis have been found to depend on the hypusination state of eIF5A, both in β cells and in immune cells. Translation of proinflammatory effector genes, such as iNOS, which is linked to T1D development, requires hypusination of eIF5A [132,133]. eIF5A^hyp^-dependent iNOS translation was linked to cytokine-induced β-cell death in both cultured human cells and in HFD-fed mice [134]. Additionally, islet inflammation in diabetic mouse models is consistently attenuated with GC7 treatment [16,26,27]. Apart from the translation of stress and inflammation-related mRNAs, another mechanism of inflammation includes the association of eIF5A^hyp^ with the nuclear exportins [135]. Specifically, in β cells, it was demonstrated that eIF5A^hyp^ is essential for nuclear export of iNOS-encoding mRNA, a process that involves the export protein exportin1 [16]. Thus, detailed insight into the role of eIF5A^hyp^ in facilitating export of nuclear mRNA encoding diabetogenic cytokines must be explored in both β cells and immune cells.

eIF5A^hyp^ has been shown to participate in the translation of immune response factors in macrophages during infection, a principle that has been recently extended to the development of islet inflammation in diabetes [136,137]. A recent study of adipose tissue macrophages, which are instrumental in the progression of obesity-induced diabetes, found that these cells expressed more eIF5A^hyp^ and exhibited enhanced DHPS activity in obese mice compared to control mice. eIF5A^hyp^ dependent transcripts include M1 proinflammatory hallmarks such as NF-κB signaling targets. Importantly, this study used a genetic knockout of DHPS in mouse macrophages [24]. Conversely, another study found that DHPS inhibition in bone marrow-derived macrophages (BMDMs) leads to decreased markers of M2 anti-inflammatory activation. However, this experiment used GC7 pharmacological inhibition of DHPS [138]. This result was supported by a study that found increased eIF5A^hyp^ in BMDMs treated with IL-4 compared to those treated with IFNγ and LPS [124]. The disparity between these studies could be the result of the documented off-target effects of GC7 [139], or differences between in vivo and in vitro experimental models, raising the need for more investigation of the role of DHPS activity in macrophage polarization. This is important for the further understanding of both major types of diabetes pathogenesis, as proinflammatory polarization of macrophages is also observed in the development of T1D [140,141]. Studying the role of eIF5A^hyp^ in macrophages, specifically in the different animal models of T1D, will highlight the role of the myeloid cell-specific eIF5A proteome in the disease progression.

In addition to macrophages, eIF5A and hypusine have been implicated in T cell dynamics during T1D pathogenesis. CD4+ Th1 cells are a major source of secreted proinflammatory cytokines, including interferon-γ (IFNγ), which is involved in the pathogenesis of both T1D and T2D [142]. One study showed that eIF5A^hyp^ was increased in CD4+ activated T cells [86]. Furthermore, in vitro inhibition of DHPS with GC7 treatment led to a significant impairment in the proliferation and proinflammatory polarization of Th1 immune cells [27]. These findings were corroborated by an in vivo humanized NOD mouse model with the expression of human GAD65 (a known major autoantigen in T1D) in β cells and human major histocompatibility complex II (MHC-II) expressed in antigen-presenting cells. In this study, GC7 treatment resulted in a decrease in pancreatic Th1 cells and a concurrent increase in pancreatic anti-inflammatory Treg cells, a result which is linked to a reduction in serum GAD65 antibody concentration and an overall delay in T1D onset [26]. These studies suggest that the role of eIF5A^hyp^ is not only restricted to β cells, but that eIF5A^hyp^ also plays a role in immune cells. To this end, further studies using cell-specific deletion models are needed to dissect the role of eIF5A^hyp^ in these individual players involved in diabetes pathogenesis.

### 3.4. Future Considerations

The importance of hypusine and eIF5A in the development of diabetes is a burgeoning field of study. Many avenues of investigation are still open and worth pursuing to tease out the details of these interactions and improve the understanding of how hypusine influences the diabetic phenotype. Because eIF5A^hyp^ is crucial during embryonic development, the generation of transgenic models to study DHPS and eIF5A has presented an obstacle [143]. Overcoming this challenge is crucial, as examining the tissue-specific loss of DHPS and eIF5A will be important in distinguishing the effects of eIF5A hypusination in each distinct cell type involved in diabetes pathogenesis. One of the engineered solutions to counter early embryonic lethality was demonstrated recently by knocking out DHPS using tamoxifen-inducible, tissue-specific Cre driver mouse models [14]. With these models, it is possible to achieve knockout past the developmental age of the tissue of interest. Future studies examining inducible DHPS and eIF5A knockouts in immune cells such as T cells will be necessary to bolster current data from GC7-based immunological studies. One aspect of eIF5A in diabetes that is understudied is that of a potential role for the un-hypusinated (eIF5A^lys^) isoform of the protein. Current literature attributes the effects of DHPS inhibition solely to the lack of hypusinated eIF5A, in line with the conventional notion that eIF5A^lys^ is inactive with no distinct function. Investigation of the potential role of un-hypusinated eIF5A in the β cell during metabolic stress is warranted.

## 4. Conclusions

In conclusion, polyamines are essential molecules for the normal functioning of β cells. However, a balance must be maintained as both deficiency and overabundance of the polyamine levels have been associated with diabetes pathogenesis and deleterious impacts on β cells (Table 1). The consequences of tipping this balance are still being investigated. Finally, inhibition of the polyamines-hypusine pathway has a protective impact in preclinical models of diabetes. It is worth noting that most of these studies have been performed using inhibitors that can have off-target effects. Therefore, more studies with genetic models, including cell-specific deletion of the enzymes involved in the polyamines-hypusine pathway, are warranted to decipher precise mechanisms and factors that render them pathogenic in diabetes and ultimately develop effective targeted therapeutics.

## Figures and Tables

**Table 1:** Summary of studies on the role of the Polyamines-Hypusine circuit in diabetes. DFMO—difluoromethylornithine; GC7—N1-guanyl-1,7-diaminoheptane; ODC—ornithine decarboxylase; DHPS—deoxyhypusine synthase; T1D—Type 1 Diabetes; T2D—Type 2 diabetes; HFD—High fat diet; STZ—Streptozotocin.

Role	References	Inhibitor	Model	Key Findings
β-cell function	Robertson et al., 2020 [13]	DFMO (inhibits ODC)	Zebrafish	DFMO promoted β-cell regeneration after β-cell injury.
	Levasseur et al., 2019 [14]	-	β cell-specific *Dhps* KO mice	With HFD, mice with a β-cell knockout of *Dhps* exhibited impaired glucose tolerance and reduced insulin secretion.
	Cerrada-Gimenez et al., 2012 [15]	-	*Ssat* overexpressing mice	Depletion of spermidine and spermine levels led to impaired glucose-stimulated insulin secretion.
Type 1 diabetes	Maier et al., 2010 [16]	-	STZ-treated mice	siRNA knockdown of *Eif5a* prevented hyperglycemia and maintained insulin secretory capacity in diabetic mice.
	Tersey et al., 2014 [17]	DFMO (inhibits ODC)	NOD mice	Inhibition of polyamine biosynthesis significantly delayed T1D incidence, with reduced insulitis.
	Bjelakovic et al., 2010 [18]	-	Human patients with T1D	Polyamine oxidase activity was increased in T1D.
	Seghieri et al., 1990 [19]	-	Human patients with T1D	Spermidine oxidase activity was significantly lower in individuals with T1D
Obesity and Type 2 diabetes	Fernandez-Garcia 2019 [20]	-	Human patients with T2D	Serum polyamine levels were elevated in T2D subjects and positively correlated with glycosylated Hb and fasting insulin.
	Robbins et al., 2010 [21]	GC7 (inhibits DHPS)	db/db Mice	Treatment with GC7 resulted in improved glucose tolerance and insulin secretion.
	Fernández et al., 2017 [22]	-	HFD-induced obese mice	Spermidine supplementation led to a decrease in body weight, improved glucose tolerance, and enhanced insulin sensitivity.
	Sadasivan et al., 2014 [23]	-	HFD-induced obese mice	Exogenous spermine decreased body weight and fasting glucose and improved glucose tolerance in obese mice.
Diabetic immunity	Anderson-Baucum et al., 2021 [24]	-	Myeloid-specific *Dhps* KO mice	eIF5A^hyp^ promoted M1 polarization and migration of macrophages in obese mice.
	Karacay et al., 2022 [25]	-	NOD mice	Spermidine supplementation increased diabetes incidence with an increased proportion of pro-inflammatory T-cells.
	Imam et al., 2019 [26]	GC7 (inhibits DHPS)	NOD mice	GC7 treatment reduced pancreatic Th1 cells and increased Treg cells, resulting in overall delay of T1D onset.
	Colvin et al., 2013 [27]	GC7 (inhibits DHPS)	NOD mice	Inhibition of DHPS led to an impairment in proliferation and proinflammatory polarization of Th1 immune cells.

## References

[1] Cerf M.E. (2013). Beta Cell Dysfunction and Insulin Resistance. Front. Endocrinol..

[2] Sims E.K., Carr A.L.J., Oram R.A., DiMeglio L.A., Evans-Molina C. (2021). 100 Years of Insulin: Celebrating the Past, Present and Future of Diabetes Therapy. Nat. Med..

[3] Saisho Y. (2015). β-Cell Dysfunction: Its Critical Role in Prevention and Management of Type 2 Diabetes. World J. Diabetes.

[4] Teodoro J.S., Nunes S., Rolo A.P., Reis F., Palmeira C.M. (2019). Therapeutic Options Targeting Oxidative Stress, Mitochondrial Dysfunction and Inflammation to Hinder the Progression of Vascular Complications of Diabetes. Front. Physiol..

[5] Burgos-Morón E., Abad-Jiménez Z., Martínez de Marañón A., Iannantuoni F., Escribano-López I., López-Domènech S., Salom C., Jover A., Mora V., Roldan I. (2019). Relationship between Oxidative Stress, ER Stress, and Inflammation in Type 2 Diabetes: The Battle Continues. J. Clin. Med..

[6] Demirtas L., Guclu A., Erdur F.M., Akbas E.M., Ozcicek A., Onk D., Turkmen K. (2016). Apoptosis, Autophagy & Endoplasmic Reticulum Stress in Diabetes Mellitus. Ind. J. Med. Res..

[7] Kulkarni A., Nadler J.L., Mirmira R.G., Casimiro I. (2021). Regulation of Tissue Inflammation by 12-Lipoxygenases. Biomolecules.

[8] Khin P.-P., Lee J.-H., Jun H.-S. (2021). A Brief Review of the Mechanisms of β-Cell Dedifferentiation in Type 2 Diabetes. Nutrients.

[9] Wang W., Zhang C. (2021). Targeting β-Cell Dedifferentiation and Transdifferentiation: Opportunities and Challenges. Endocr. Connect..

[10] Minois N., Carmona-Gutierrez D., Madeo F. (2011). Polyamines in Aging and Disease. Aging.

[11] Schuller A.P., Wu C.C.-C., Dever T.E., Buskirk A.R., Green R. (2017). EIF5A Functions Globally in Translation Elongation and Termination. Mol. Cell.

[12] Pelechano V., Alepuz P. (2017). EIF5A Facilitates Translation Termination Globally and Promotes the Elongation of Many Non Polyproline-Specific Tripeptide Sequences. Nucl. Acids Res..

[13] Robertson M.A., Padgett L.R., Fine J.A., Chopra G., Mastracci T.L. (2020). Targeting Polyamine Biosynthesis to Stimulate Beta Cell Regeneration in Zebrafish. Islets.

[14] Levasseur E.M., Yamada K., Piñeros A.R., Wu W., Syed F., Orr K.S., Anderson-Baucum E., Mastracci T.L., Maier B., Mosley A.L. (2019). Hypusine Biosynthesis in β Cells Links Polyamine Metabolism to Facultative Cellular Proliferation to Maintain Glucose Homeostasis. Sci. Signal..

[15] Cerrada-Gimenez M., Tusa M., Casellas A., Pirinen E., Moya M., Bosch F., Alhonen L. (2012). Altered Glucose-Stimulated Insulin Secretion in a Mouse Line with Activated Polyamine Catabolism. Transgen. Res..

[16] Maier B., Ogihara T., Trace A.P., Tersey S.A., Robbins R.D., Chakrabarti S.K., Nunemaker C.S., Stull N.D., Taylor C.A., Thompson J.E. (2010). The Unique Hypusine Modification of EIF5A Promotes Islet Beta Cell Inflammation and Dysfunction in Mice. J. Clin. Investig..

[17] Tersey S.A., Colvin S.C., Maier B., Mirmira R.G. (2014). Protective Effects of Polyamine Depletion in Mouse Models of Type 1 Diabetes: Implications for Therapy. Amino Acids.

[18] Bjelakovic G., Beninati S., Bjelakovic B., Sokolovic D., Jevtovic T., Stojanovic I., Rossi S., Tabolacci C., Kocić G., Pavlovic D. (2010). Does Polyamine Oxidase Activity Influence the Oxidative Metabolism of Children Who Suffer of Diabetes Mellitus?. Mol. Cell Biochem..

[19] Seghieri G., Gironi A., Niccolai M., Mammini P., Alviggi L., De Giorgio L.A., Caselli P., Bartolomei G. (1990). Serum Spermidine Oxidase Activity in Patients with Insulin-Dependent Diabetes Mellitus and Microvascular Complications. Acta Diabetol. Lat..

[20] Fernandez-Garcia J.C., Delpino-Rius A., Samarra I., Castellano-Castillo D., Muñoz-Garach A., Bernal-Lopez M.R., Queipo-Ortuño M.I., Cardona F., Ramos-Molina B., Tinahones F.J. (2019). Type 2 Diabetes Is Associated with a Different Pattern of Serum Polyamines: A Case–Control Study from the PREDIMED-Plus Trial. J. Clin. Med..

[21] Robbins R.D., Tersey S.A., Ogihara T., Gupta D., Farb T.B., Ficorilli J., Bokvist K., Maier B., Mirmira R.G. (2010). Inhibition of Deoxyhypusine Synthase Enhances Islet β Cell Function and Survival in the Setting of Endoplasmic Reticulum Stress and Type 2 Diabetes. J. Biol. Chem..

[22] Fernández Á.F., Bárcena C., Martínez-García G.G., Tamargo-Gómez I., Suárez M.F., Pietrocola F., Castoldi F., Esteban L., Sierra-Filardi E., Boya P. (2017). Autophagy Couteracts Weight Gain, Lipotoxicity and Pancreatic β-Cell Death upon Hypercaloric pro-Diabetic Regimens. Cell Death Dis..

[23] Sadasivan S.K., Vasamsetti B., Singh J., Marikunte V.V., Oommen A.M., Jagannath M.R., Pralhada Rao R. (2014). Exogenous Administration of Spermine Improves Glucose Utilization and Decreases Bodyweight in Mice. Eur. J. Pharm..

[24] Anderson-Baucum E., Piñeros A.R., Kulkarni A., Webb-Robertson B.-J., Maier B., Anderson R.M., Wu W., Tersey S.A., Mastracci T.L., Casimiro I. (2021). Deoxyhypusine Synthase Promotes a Pro-Inflammatory Macrophage Phenotype. Cell Metab..

[25] Karacay C., Prietl B., Harer C., Ehall B., Haudum C.W., Bounab K., Franz J., Eisenberg T., Madeo F., Kolb D. (2022). The Effect of Spermidine on Autoimmunity and Beta Cell Function in NOD Mice. Sci. Rep..

[26] Imam S., Prathibha R., Dar P., Almotah K., Al-Khudhair A., Hasan S.A.-M., Salim N., Jilani T.N., Mirmira R.G., Jaume J.C. (2019). EIF5A Inhibition Influences T Cell Dynamics in the Pancreatic Microenvironment of the Humanized Mouse Model of Type 1 Diabetes. Sci. Rep..

[27] Colvin S.C., Maier B., Morris D.L., Tersey S.A., Mirmira R.G. (2013). Deoxyhypusine Synthase Promotes Differentiation and Proliferation of T Helper Type 1 (Th1) Cells in Autoimmune Diabetes. J. Biol. Chem..

[28] van Dam L., Korolev N., Nordenskiöld L. (2002). Polyamine–Nucleic Acid Interactions and the Effects on Structure in Oriented DNA Fibers. Nucl. Acids Res..

[29] Podestà A., Indrieri M., Brogioli D., Manning G.S., Milani P., Guerra R., Finzi L., Dunlap D. (2005). Positively Charged Surfaces Increase the Flexibility of DNA. Biophys. J..

[30] Lightfoot H.L., Hall J. (2014). Endogenous Polyamine Function—The RNA Perspective. Nucl. Acids Res..

[31] Brooks W.H. (2013). Increased Polyamines Alter Chromatin and Stabilize Autoantigens in Autoimmune Diseases. Front. Immunol..

[32] Perepelytsya S., Uličný J., Laaksonen A., Mocci F. (2019). Pattern Preferences of DNA Nucleotide Motifs by Polyamines Putrescine^2+^, Spermidine^3+^ and Spermine^4+^. Nucl. Acids Res..

[33] Gerner E.W., Russell D.H. (1977). The Relationship between Polyamine Accumulation and DNA Replication in Synchronized Chinese Hamster Ovary Cells after Heat Shock. Cancer Res..

[34] Gallo C.J., Koza R.A., Herbst E.J. (1986). Polyamines and HeLa-Cell DNA Replication. Biochem. J..

[35] Tkachenko A.G., Nesterova L.Y. (2003). Polyamines as Modulators of Gene Expression under Oxidative Stress in *Escherichia coli*. Biochemistry.

[36] Sakamoto A., Terui Y., Uemura T., Igarashi K., Kashiwagi K. (2020). Polyamines Regulate Gene Expression by Stimulating Translation of Histone Acetyltransferase MRNAs. J. Biol. Chem..

[37] Matthews H.R. (1993). Polyamines, Chromatin Structure and Transcription. Bioessays.

[38] Xiao L., Rao J.N., Zou T., Liu L., Marasa B.S., Chen J., Turner D.J., Zhou H., Gorospe M., Wang J.-Y. (2007). Polyamines Regulate the Stability of Activating Transcription Factor-2 MRNA through RNA-Binding Protein HuR in Intestinal Epithelial Cells. Mol. Biol. Cell.

[39] Huang S.C., Panagiotidis C.A., Canellakis E.S. (1990). Transcriptional Effects of Polyamines on Ribosomal Proteins and on Polyamine-Synthesizing Enzymes in *Escherichia coli*. Proc. Natl. Acad. Sci. USA.

[40] Igarashi K., Kashiwagi K. (2018). Effects of Polyamines on Protein Synthesis and Growth of *Escherichia coli*. J. Biol. Chem..

[41] Ivanov I.P., Shin B.-S., Loughran G., Tzani I., Young-Baird S.K., Cao C., Atkins J.F., Dever T.E. (2018). Polyamine Control of Translation Elongation Regulates Start Site Selection on the Antizyme Inhibitor MRNA via Ribosome Queuing. Mol. Cell.

[42] Perez-Leal O., Barrero C.A., Clarkson A.B., Casero R.A., Merali S. (2012). Polyamine-Regulated Translation of Spermidine/Spermine-N1-Acetyltransferase. Mol. Cell. Biol..

[43] Thomas T., Thomas T.J. (2001). Polyamines in Cell Growth and Cell Death: Molecular Mechanisms and Therapeutic Applications. Cell Mol. Life Sci..

[44] Mohapatra S., Cherry S., Minocha R., Majumdar R., Thangavel P., Long S., Minocha S.C. (2010). The Response of High and Low Polyamine-Producing Cell Lines to Aluminum and Calcium Stress. Plant Physiol. Biochem..

[45] Kahana C. (2009). Regulation of Cellular Polyamine Levels and Cellular Proliferation by Antizyme and Antizyme Inhibitor. Essays Biochem..

[46] Whelly S.M. (1991). Role of Polyamine in the Regulation of RNA Synthesis in Uterine Nucleoli. J. Steroid. Biochem. Mol. Biol..

[47] Terui Y., Tabei Y., Akiyama M., Higashi K., Tomitori H., Yamamoto K., Ishihama A., Igarashi K., Kashiwagi K. (2010). Ribosome Modulation Factor, an Important Protein for Cell Viability Encoded by the Polyamine Modulon. J. Biol. Chem..

[48] Rato C., Amirova S.R., Bates D.G., Stansfield I., Wallace H.M. (2011). Translational Recoding as a Feedback Controller: Systems Approaches Reveal Polyamine-Specific Effects on the Antizyme Ribosomal Frameshift. Nucl. Acids Res..

[49] McMurry L.M., Algranati I.D. (1986). Effect of Polyamines on Translation Fidelity in Vivo. Eur. J. Biochem..

[50] Landau G., Bercovich Z., Park M.H., Kahana C. (2010). The Role of Polyamines in Supporting Growth of Mammalian Cells Is Mediated through Their Requirement for Translation Initiation and Elongation. J. Biol. Chem..

[51] Park M.H., Wolff E.C. (2018). Hypusine, a Polyamine-Derived Amino Acid Critical for Eukaryotic Translation. J. Biol. Chem..

[52] Muñoz-Esparza N.C., Latorre-Moratalla M.L., Comas-Basté O., Toro-Funes N., Veciana-Nogués M.T., Vidal-Carou M.C. (2019). Polyamines in Food. Front. Nutr..

[53] Tofalo R., Cocchi S., Suzzi G. (2019). Polyamines and Gut Microbiota. Front Nutr..

[54] Seiler N. (1990). Polyamine Metabolism. Digestion.

[55] Wu G., Morris S.M. (1998). Arginine Metabolism: Nitric Oxide and Beyond. Biochem. J..

[56] Jackson L.K., Brooks H.B., Myers D.P., Phillips M.A. (2003). Ornithine Decarboxylase Promotes Catalysis by Binding the Carboxylate in a Buried Pocket Containing Phenylalanine 397. Biochemistry.

[57] Tabor C.W., Tabor H. (1984). Polyamines. Annu. Rev. Biochem..

[58] Wu H.-Y., Chen S.-F., Hsieh J.-Y., Chou F., Wang Y.-H., Lin W.-T., Lee P.-Y., Yu Y.-J., Lin L.-Y., Lin T.-S. (2015). Structural Basis of Antizyme-Mediated Regulation of Polyamine Homeostasis. Proc. Natl. Acad. Sci. USA.

[59] Pegg A.E. (2009). S-Adenosylmethionine Decarboxylase. Essays Biochem..

[60] Casero R.A., Pegg A.E. (2009). Polyamine Catabolism and Disease. Biochem. J..

[61] Vujcic S., Diegelman P., Bacchi C.J., Kramer D.L., Porter C.W. (2002). Identification and Characterization of a Novel Flavin-Containing Spermine Oxidase of Mammalian Cell Origin. Biochem. J..

[62] Sessa A., Perin A. (1994). Diamine Oxidase in Relation to Diamine and Polyamine Metabolism. Agents Act..

[63] Mitchell J.L., Judd G.G., Leyser A., Choe C. (1998). Osmotic Stress Induces Variation in Cellular Levels of Ornithine Decarboxylase-Antizyme. Biochem. J..

[64] Ray R.M., Viar M.J., Patel T.B., Johnson L.R. (1999). Interaction of Asparagine and EGF in the Regulation of Ornithine Decarboxylase in IEC-6 Cells. Am. J. Physiol..

[65] Rinehart C.A., Viceps-Madore D., Fong W.F., Ortiz J.G., Canellakis E.S. (1985). The Effect of Transport System A and N Amino Acids and of Nerve and Epidermal Growth Factors on the Induction of Ornithine Decarboxylase Activity. J. Cell Physiol..

[66] Ramos-Molina B., Lambertos A., Peñafiel R. (2018). Antizyme Inhibitors in Polyamine Metabolism and Beyond: Physiopathological Implications. Med. Sci..

[67] Hill J.R., Morris D.R. (1993). Cell-Specific Translational Regulation of S-Adenosylmethionine Decarboxylase MRNA. Dependence on Translation and Coding Capacity of the Cis-Acting Upstream Open Reading Frame. J. Biol. Chem..

[68] Casero R.A., Pegg A.E. (1993). Spermidine/Spermine N1-Acetyltransferase--the Turning Point in Polyamine Metabolism. FASEB J..

[69] Pegg A.E. (2008). Spermidine/Spermine-N(1)-Acetyltransferase: A Key Metabolic Regulator. Am. J. Physiol. Endocr. Metab..

[70] Abdulhussein A.A., Wallace H.M. (2014). Polyamines and Membrane Transporters. Amino Acids.

[71] Uemura T., Gerner E.W. (2011). Polyamine Transport Systems in Mammalian Cells and Tissues. Methods Mol. Biol..

[72] Hougaard D.M., Larsson L.I. (1986). Localization and Possible Function of Polyamines in Protein and Peptide Secreting Cells. Med. Biol..

[73] Hougaard D.M., Nielsen J.H., Larsson L.I. (1986). Localization and Biosynthesis of Polyamines in Insulin-Producing Cells. Biochem. J..

[74] Mastracci T.L., Robertson M.A., Mirmira R.G., Anderson R.M. (2015). Polyamine Biosynthesis Is Critical for Growth and Differentiation of the Pancreas. Sci. Rep..

[75] Sjöholm A. (1993). Role of Polyamines in the Regulation of Proliferation and Hormone Production by Insulin-Secreting Cells. Am. J. Physiol..

[76] Sjöholm A., Arkhammar P., Berggren P.O., Andersson A. (2001). Polyamines in Pancreatic Islets of Obese-Hyperglycemic (Ob/Ob) Mice of Different Ages. Am. J. Physiol. Cell Physiol..

[77] Yang B., Covington B.A., Chen W. (2020). In Vivo Generation and Regeneration of β Cells in Zebrafish. Cell Regen..

[78] Moro E., Gnügge L., Braghetta P., Bortolussi M., Argenton F. (2009). Analysis of Beta Cell Proliferation Dynamics in Zebrafish. Dev. Biol..

[79] Kulkarni A.A., Conteh A.M., Sorrell C.A., Mirmira A., Tersey S.A., Mirmira R.G., Linnemann A.K., Anderson R.M. (2018). An In Vivo Zebrafish Model for Interrogating ROS-Mediated Pancreatic β-Cell Injury, Response, and Prevention. Oxid. Med. Cell Longev..

[80] Hernandez-Perez M., Kulkarni A., Samala N., Sorrell C., El K., Haider I., Mukhtar Aleem A., Holman T.R., Rai G., Tersey S.A. (2020). A 12-Lipoxygenase-Gpr31 Signaling Axis Is Required for Pancreatic Organogenesis in the Zebrafish. FASEB J..

[81] Welsh N., Sjöholm A. (1988). Polyamines and Insulin Production in Isolated Mouse Pancreatic Islets. Biochem. J..

[82] Welsh N. (1990). A Role for Polyamines in Glucose-Stimulated Insulin-Gene Expression. Biochem. J..

[83] Pirinen E., Kuulasmaa T., Pietilä M., Heikkinen S., Tusa M., Itkonen P., Boman S., Skommer J., Virkamäki A., Hohtola E. (2007). Enhanced Polyamine Catabolism Alters Homeostatic Control of White Adipose Tissue Mass, Energy Expenditure, and Glucose Metabolism. Mol. Cell. Biol..

[84] Yuan F., Zhang L., Cao Y., Gao W., Zhao C., Fang Y., Zahedi K., Soleimani M., Lu X., Fang Z. (2018). Spermidine/Spermine N1-Acetyltransferase-Mediated Polyamine Catabolism Regulates Beige Adipocyte Biogenesis. Metabolism.

[85] Niiranen K., Keinänen T.A., Pirinen E., Heikkinen S., Tusa M., Fatrai S., Suppola S., Pietilä M., Uimari A., Laakso M. (2006). Mice with Targeted Disruption of Spermidine/Spermine N1-Acetyltransferase Gene Maintain Nearly Normal Tissue Polyamine Homeostasis but Show Signs of Insulin Resistance upon Aging. J. Cell. Mol. Med..

[86] Puleston D.J., Baixauli F., Sanin D.E., Edwards-Hicks J., Villa M., Kabat A.M., Kamiński M.M., Stanckzak M., Weiss H.J., Grzes K.M. (2021). Polyamine Metabolism Is a Central Determinant of Helper T Cell Lineage Fidelity. Cell.

[87] Tsalamandris S., Antonopoulos A.S., Oikonomou E., Papamikroulis G.-A., Vogiatzi G., Papaioannou S., Deftereos S., Tousoulis D. (2019). The Role of Inflammation in Diabetes: Current Concepts and Future Perspectives. Eur. Cardiol..

[88] Piñeros A., Kulkarni A., Gao H., Orr K., Glenn L., Huang F., Liu Y., Gannon M., Syed F., Wu W. (2021). Proinflammatory Signaling in Islet β Cells Propagates Invasion of Pathogenic Immune Cells in Autoimmune Diabetes.

[89] Marro B.S., Legrain S., Ware B.C., Oldstone M.B.A. (2019). Macrophage IFN-I Signaling Promotes Autoreactive T Cell Infiltration into Islets in Type 1 Diabetes Model. JCI Insight.

[90] Kulkarni A., Pineros A.R., Walsh M.A., Casimiro I., Ibrahim S., Hernandez-Perez M., Orr K.S., Glenn L., Nadler J.L., Morris M.A. (2021). 12-Lipoxygenase Governs the Innate Immune Pathogenesis of Islet Inflammation and Autoimmune Diabetes. JCI Insight.

[91] Davanso M.R., Crisma A.R., Braga T.T., Masi L.N., do Amaral C.L., Leal V.N.C., de Lima D.S., Patente T.A., Barbuto J.A., Corrêa-Giannella M.L. (2020). Macrophage Inflammatory State in Type 1 Diabetes: Triggered by NLRP3/INOS Pathway and Attenuated by Docosahexaenoic Acid (DHA). Clin. Sci..

[92] Mensah-Brown E., Shahin A., Parekh K., Hakim A.A., Shamisi M.A., Hsu D.K., Lukic M.L. (2006). Functional Capacity of Macrophages Determines the Induction of Type 1 Diabetes. Ann. N. Y. Acad. Sci..

[93] Tesch G.H. (2007). Role of Macrophages in Complications of Type 2 Diabetes. Clin. Exp. Pharm. Physiol..

[94] Roep B.O. (2003). The Role of T-Cells in the Pathogenesis of Type 1 Diabetes: From Cause to Cure. Diabetologia.

[95] Tsai S., Shameli A., Santamaria P. (2008). CD8+ T Cells in Type 1 Diabetes. Adv. Immunol..

[96] Kent S.C., Mannering S.I., Michels A.W., Babon J.A.B. (2017). Deciphering the Pathogenesis of Human Type 1 Diabetes (T1D) by Interrogating T Cells from the Scene of the Crime. Curr. Diab. Rep..

[97] Hull C.M., Peakman M., Tree T.I.M. (2017). Regulatory T Cell Dysfunction in Type 1 Diabetes: What’s Broken and How Can We Fix It?. Diabetologia.

[98] Xia C., Rao X., Zhong J. (2017). Role of T Lymphocytes in Type 2 Diabetes and Diabetes-Associated Inflammation. J. Diabetes Res..

[99] Lau E.Y.M., Carroll E.C., Callender L.A., Hood G.A., Berryman V., Pattrick M., Finer S., Hitman G.A., Ackland G.L., Henson S.M. (2019). Type 2 Diabetes Is Associated with the Accumulation of Senescent T Cells. Clin. Exp. Immunol..

[100] Latour Y.L., Gobert A.P., Wilson K.T. (2020). The Role of Polyamines in the Regulation of Macrophage Polarization and Function. Amino Acids.

[101] Wang Z. (2021). Polyamines Instruct T-Cell Differentiation. Nat. Cell Biol..

[102] Sandler S., Bendtzen K., Eizirik D.L., Sjöholm A., Welsh N. (1989). Decreased Cell Replication and Polyamine Content in Insulin-Producing Cells after Exposure to Human Interleukin 1 Beta. Immunol. Lett..

[103] Smismans A., Eizirik D.L., Pipeleers D.G. (2000). Interleukin-1beta Induces Ornithine Decarboxylase Activity in Insulin-Producing Cells. Cytokine.

[104] Ohtani M., Mizuno I., Kojima Y., Ishikawa Y., Sodeno M., Asakura Y., Samejima K., Oka T. (2009). Spermidine Regulates Insulin Synthesis and Cytoplasmic Ca^2+^ in Mouse Beta-TC6 Insulinoma Cells. Cell Struct. Funct..

[105] Lenzen S., Rustenbeck I. (1991). Effects of IP3, Spermine, and Mg^2+^ on Regulation of Ca^2+^ Transport by Endoplasmic Reticulum and Mitochondria in Permeabilized Pancreatic Islets. Diabetes.

[106] Yong J., Johnson J.D., Arvan P., Han J., Kaufman R.J. (2021). Therapeutic Opportunities for Pancreatic β-Cell ER Stress in Diabetes Mellitus. Nat. Rev. Endocrinol..

[107] Codoñer-Franch P., Tavárez-Alonso S., Murria-Estal R., Herrera-Martín G., Alonso-Iglesias E. (2011). Polyamines Are Increased in Obese Children and Are Related to Markers of Oxidative/Nitrosative Stress and Angiogenesis. J. Clin. Endocrinol. Metab..

[108] Marselli L., Bosi E., De Luca C., Del Guerra S., Tesi M., Suleiman M., Marchetti P. (2021). Arginase 2 and Polyamines in Human Pancreatic Beta Cells: Possible Role in the Pathogenesis of Type 2 Diabetes. Int. J. Mol. Sci..

[109] Hesterberg R.S., Cleveland J.L., Epling-Burnette P.K. (2018). Role of Polyamines in Immune Cell Functions. Med. Sci..

[110] Gao M., Zhao W., Li C., Xie X., Li M., Bi Y., Fang F., Du Y., Liu X. (2018). Spermidine Ameliorates Non-Alcoholic Fatty Liver Disease through Regulating Lipid Metabolism via AMPK. Biochem. Biophys. Res. Commun..

[111] Méndez J.D., Balderas F.L. (2006). Inhibition by L-Arginine and Spermidine of Hemoglobin Glycation and Lipid Peroxidation in Rats with Induced Diabetes. Biomed. Pharm..

[112] Méndez J.D., Hernández R.D.H. (2005). L-Arginine and Polyamine Administration Protect Beta-Cells against Alloxan Diabetogenic Effect in Sprague-Dawley Rats. Biomed. Pharm..

[113] Jafarnejad A., Bathaie S.Z., Nakhjavani M., Hassan M.Z. (2008). Effect of Spermine on Lipid Profile and HDL Functionality in the Streptozotocin-Induced Diabetic Rat Model. Life Sci..

[114] Fetterman J.L., Holbrook M., Flint N., Feng B., Bretón-Romero R., Linder E.A., Berk B.D., Duess M.-A., Farb M.G., Gokce N. (2016). Restoration of Autophagy in Endothelial Cells from Patients with Diabetes Mellitus Improves Nitric Oxide Signaling. Atherosclerosis.

[115] Gutierrez E., Shin B.-S., Woolstenhulme C.J., Kim J.-R., Saini P., Buskirk A.R., Dever T.E. (2013). EIF5A Promotes Translation of Polyproline Motifs. Mol. Cell.

[116] Park J.H., Wolff E.C., Park M.H. (2011). Assay of Deoxyhypusine Hydroxylase Activity. Methods Mol. Biol..

[117] Clement P.M.J., Johansson H.E., Wolff E.C., Park M.H. (2006). Differential Expression of EIF5A-1 and EIF5A-2 in Human Cancer Cells. FEBS J..

[118] Benne R., Hershey J.W. (1978). The Mechanism of Action of Protein Synthesis Initiation Factors from Rabbit Reticulocytes. J. Biol. Chem..

[119] Saini P., Eyler D.E., Green R., Dever T.E. (2009). Hypusine-Containing Protein EIF5A Promotes Translation Elongation. Nature.

[120] Park M.H., Wolff E.C., Smit-McBride Z., Hershey J.W., Folk J.E. (1991). Comparison of the Activities of Variant Forms of EIF-4D. The Requirement for Hypusine or Deoxyhypusine. J. Biol. Chem..

[121] Park M.H., Nishimura K., Zanelli C.F., Valentini S.R. (2010). Functional Significance of EIF5A and Its Hypusine Modification in Eukaryotes. Amino Acids.

[122] Mastracci T.L., Colvin S.C., Padgett L.R., Mirmira R.G. (2020). Hypusinated EIF5A Is Expressed in the Pancreas and Spleen of Individuals with Type 1 and Type 2 Diabetes. PLoS ONE.

[123] Padgett L.R., Robertson M.A., Anderson-Baucum E.K., Connors C.T., Wu W., Mirmira R.G., Mastracci T.L. (2021). Deoxyhypusine Synthase, an Essential Enzyme for Hypusine Biosynthesis, Is Required for Proper Exocrine Pancreas Development. FASEB J..

[124] Nakamura A., Kurihara S., Takahashi D., Ohashi W., Nakamura Y., Kimura S., Onuki M., Kume A., Sasazawa Y., Furusawa Y. (2021). Symbiotic Polyamine Metabolism Regulates Epithelial Proliferation and Macrophage Differentiation in the Colon. Nat. Commun..

[125] Nishimura K., Murozumi K., Shirahata A., Park M.H., Kashiwagi K., Igarashi K. (2005). Independent Roles of EIF5A and Polyamines in Cell Proliferation. Biochem. J..

[126] Saisho Y., Butler A.E., Manesso E., Elashoff D., Rizza R.A., Butler P.C. (2013). β-Cell Mass and Turnover in Humans: Effects of Obesity and Aging. Diabetes Care.

[127] Mosser R.E., Maulis M.F., Moullé V.S., Dunn J.C., Carboneau B.A., Arasi K., Pappan K., Poitout V., Gannon M. (2015). High-Fat Diet-Induced β-Cell Proliferation Occurs Prior to Insulin Resistance in C57Bl/6J Male Mice. Am. J. Physiol. Endocrinol. Metab..

[128] Stamateris R.E., Sharma R.B., Hollern D.A., Alonso L.C. (2013). Adaptive β-Cell Proliferation Increases Early in High-Fat Feeding in Mice, Concurrent with Metabolic Changes, with Induction of Islet Cyclin D2 Expression. Am. J. Physiol. Endocrinol. Metab..

[129] Keenan H.A., Sun J.K., Levine J., Doria A., Aiello L.P., Eisenbarth G., Bonner-Weir S., King G.L. (2010). Residual Insulin Production and Pancreatic SS-Cell Turnover after 50 Years of Diabetes: Joslin Medalist Study. Diabetes.

[130] Sreenan S., Pick A.J., Levisetti M., Baldwin A.C., Pugh W., Polonsky K.S. (1999). Increased Beta-Cell Proliferation and Reduced Mass before Diabetes Onset in the Nonobese Diabetic Mouse. Diabetes.

[131] Dirice E., Kahraman S., De Jesus D.F., El Ouaamari A., Basile G., Baker R.L., Yigit B., Piehowski P.D., Kim M.-J., Dwyer A.J. (2019). Increased β-Cell Proliferation before Immune Cell Invasion Prevents Progression of Type 1 Diabetes. Nat. Metab..

[132] Johannesen J., Pie A., Pociot F., Kristiansen O.P., Karlsen A.E., Nerup J., Danish Study Group of Diabetes in Childhood (2001). The Danish Insulin-dependent Diabetes Mellitus Epidemiology and Genetics Group Linkage of the Human Inducible Nitric Oxide Synthase Gene to Type 1 Diabetes. J. Clin. Endocrinol. Metab..

[133] Templin A.T., Maier B., Nishiki Y., Tersey S.A., Mirmira R.G. (2011). Deoxyhypusine Synthase Haploinsufficiency Attenuates Acute Cytokine Signaling. Cell Cycle.

[134] Turpaev K., Krizhanovskii C., Wang X., Sargsyan E., Bergsten P., Welsh N. (2019). The Protein Synthesis Inhibitor Brusatol Normalizes High-Fat Diet-Induced Glucose Intolerance in Male C57BL/6 Mice: Role of Translation Factor EIF5A Hypusination. FASEB J..

[135] Rosorius O., Reichart B., Krätzer F., Heger P., Dabauvalle M.C., Hauber J. (1999). Nuclear Pore Localization and Nucleocytoplasmic Transport of EIF-5A: Evidence for Direct Interaction with the Export Receptor CRM1. J. Cell Sci..

[136] Gobert A.P., Finley J.L., Latour Y.L., Asim M., Smith T.M., Verriere T.G., Barry D.P., Allaman M.M., Delagado A.G., Rose K.L. (2020). Hypusination Orchestrates the Antimicrobial Response of Macrophages. Cell Rep..

[137] de Almeida O.P., Toledo T.R., Rossi D., de Rossetto D.B., Watanabe T.F., Galvão F.C., Medeiros A.I., Zanelli C.F., Valentini S.R. (2014). Hypusine Modification of the Ribosome-Binding Protein EIF5A, a Target for New Anti-Inflammatory Drugs: Understanding the Action of the Inhibitor GC7 on a Murine Macrophage Cell Line. Curr. Pharm. Des..

[138] Puleston D.J., Buck M.D., Klein Geltink R.I., Kyle R.L., Caputa G., O’Sullivan D., Cameron A.M., Castoldi A., Musa Y., Kabat A.M. (2019). Polyamines and EIF5A Hypusination Modulate Mitochondrial Respiration and Macrophage Activation. Cell Metab..

[139] Oliverio S., Corazzari M., Sestito C., Piredda L., Ippolito G., Piacentini M. (2014). The Spermidine Analogue GC7 (N1-Guanyl-1,7-Diamineoheptane) Induces Autophagy through a Mechanism Not Involving the Hypusination of EIF5A. Amino Acids.

[140] Wang F., Sun F., Luo J., Yue T., Chen L., Zhou H., Zhang J., Yang C., Luo X., Zhou Q. (2019). Loss of Ubiquitin-Conjugating Enzyme E2 (Ubc9) in Macrophages Exacerbates Multiple Low-Dose Streptozotocin-Induced Diabetes by Attenuating M2 Macrophage Polarization. Cell Death Dis..

[141] Calderon B., Suri A., Unanue E.R. (2006). In CD4+ T-Cell-Induced Diabetes, Macrophages Are the Final Effector Cells That Mediate Islet Beta-Cell Killing: Studies from an Acute Model. Am. J. Pathol..

[142] Tsiavou A., Hatziagelaki E., Chaidaroglou A., Koniavitou K., Degiannis D., Raptis S.A. (2005). Correlation between Intracellular Interferon-Gamma (IFN-Gamma) Production by CD4+ and CD8+ Lymphocytes and IFN-Gamma Gene Polymorphism in Patients with Type 2 Diabetes Mellitus and Latent Autoimmune Diabetes of Adults (LADA). Cytokine.

[143] Nishimura K., Lee S.B., Park J.H., Park M.H. (2012). Essential Role of EIF5A-1 and Deoxyhypusine Synthase in Mouse Embryonic Development. Amino Acids.

